# Laparoscopic total gastrectomy performed for juvenile polyposis of the stomach: A case report

**DOI:** 10.1016/j.ijscr.2022.107368

**Published:** 2022-06-29

**Authors:** Misato Ito, Hisashi Onozawa, Masaru Saito, Hirofumi Ami, Shinji Ohki, Yoshihisa Koyama, Kono Koji

**Affiliations:** a Department of Gastrointestinal Tract Surgery, Fukushima medical university, Fukushima, Japan; b Department of Surgery, Ohara General Hospital, Fukushima, Japan

**Keywords:** Juvenile polyposis of the stomach, Laparoscopic surgery, Total gastrectomy

## Abstract

**Introduction and importance:**

Juvenile polyposis of the stomach (JPST) is a very rare disease and has been reported to have malignant potential. Total gastrectomy has been recommended as a standard treatment. Recently, the usefulness of laparoscopic surgery for this disease has been reported; however, in laparoscopic surgery, maintaining the surgical space is difficult because of the distended and thickened stomach wall that polyposis causes.

**Case presentation:**

A 64-year-old woman was admitted to our hospital because she became malnourished due to loss of appetite. She had no family history of gastrointestinal polyposis and was diagnosed with gastric polyposis and polyp-related anemia eight years previously. She received endoscopic submucosal dissection of early gastric cancer twice in another hospital. Thereafter, the patient received an annual upper gastrointestinal endoscopy and took iron supplements for anemia due to occasional bleeding from polyps. However, the number of polyps increased over time. Enhanced computed tomography showed gastric wall thickening and multiple gastric polyps. She was diagnosed as having JPST and underwent laparoscopic total gastrectomy. She was discharged on postoperative Day 10.

**Clinical discussion:**

In the present case, similar to previous cases, standard laparoscopic surgery could be performed although the patient had excessive distention and congestion of the stomach. This report suggests that laparoscopic surgery is a safe and feasible option for patients with JPST and is preferable because of better cosmetic effects, especially for young female patients.

**Conclusion:**

We successfully performed laparoscopic surgery to treat a rare case of JPST.

## Highlights

• A rare case of juvenile polyposis of the stomach (JPST), for which laparoscopic surgery was performed.
• Laparoscopic surgery is preferable because of better cosmetic effects, especially for young female patients.
• JPST has malignant potential of gastric cancer, and JPST patients are needed for careful follow-up.

## 1 Introduction

Juvenile polyposis syndrome (JPS) is a gastrointestinal polyposis characterized by the development of numerous hamartomatous and nonneoplastic polyps, and was reported the presence of colorectal, gastric, and small bowel juvenile polyps in 98 %, 14 %, and 8.8 %, respectively [1]. Juvenile polyposis of the stomach (JPST) is a very rare disease, and its malignant potential has been reported previously [2]. Total gastrectomy is considered to be the standard treatment [3]. Recently, the usefulness of laparoscopic surgery for JPST has been reported; however, in case of laparoscopic surgery, maintaining the surgical space is difficult, because of the distended and thickened stomach wall that polyposis causes. Here, we report a case of JPST, which was successfully treated by laparoscopic total gastrectomy. This case report has been reported in line with the SCARE 2020 criteria [4].

## 2 Case presentation

**Fig. 1:**
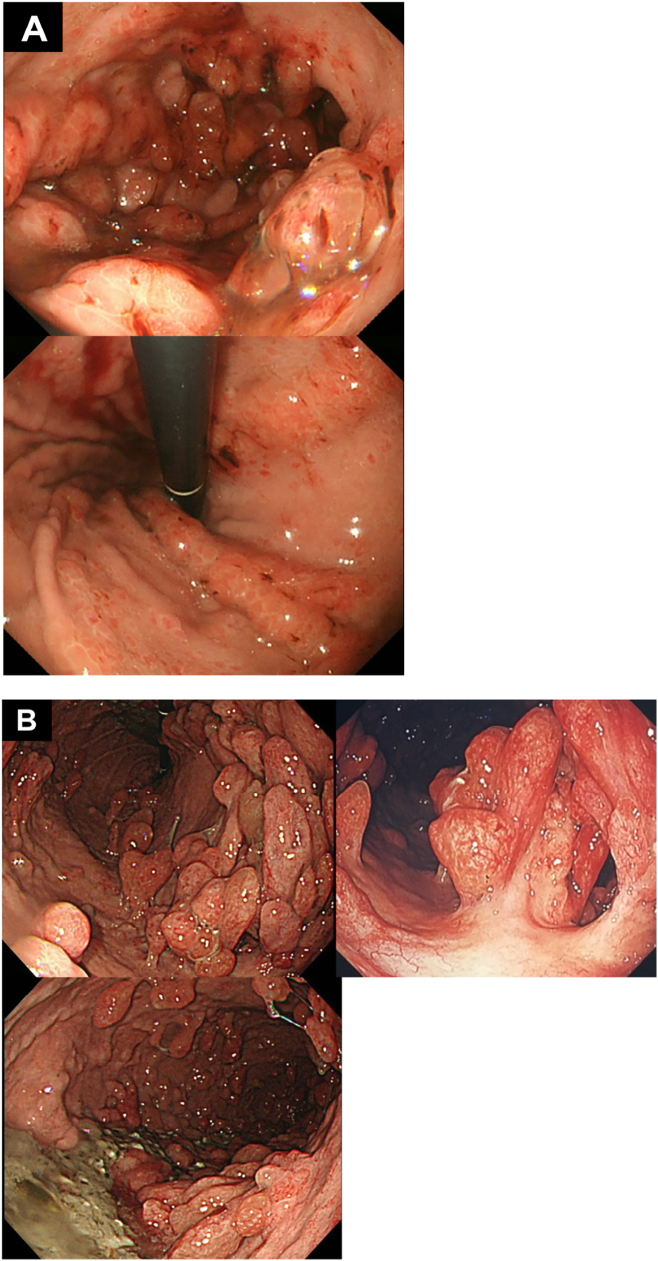
Upper gastrointestinal endoscopy images taken 8 years previously (A) and on admission to our hospital (B). A significant increase in the size and number of polyps was observed. Key words: Juvenile gastric polyposis, laparoscopic surgery, total gastrectomy.

**Fig. 2:**
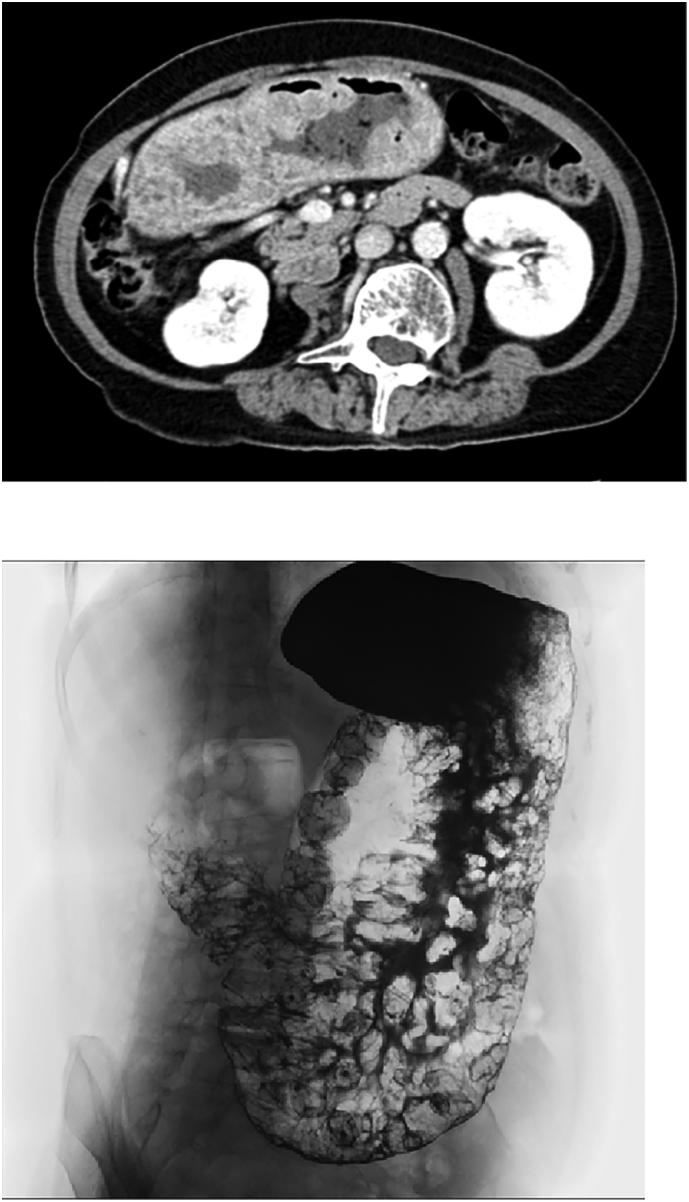
Enhanced computed tomography showed gastric wall thickening and multiple gastric polyps without lymphadenopathy or distant metastasis (A). Upper gastrointestinal series revealed that multiple polypoid lesions were widespread throughout the entire stomach except for the lesser curvature and the fundus (B).

**Fig. 3:**
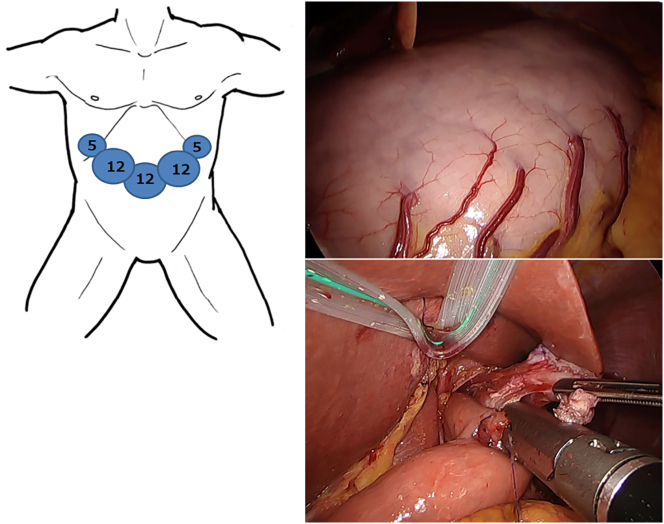
Laparoscopic total gastrectomy was performed with Roux-en-Y esophagojejunostomy by overlap method. Although excessive distention and congestion of the stomach were observed, standard laparoscopic surgery could be performed.

**Fig. 4:**
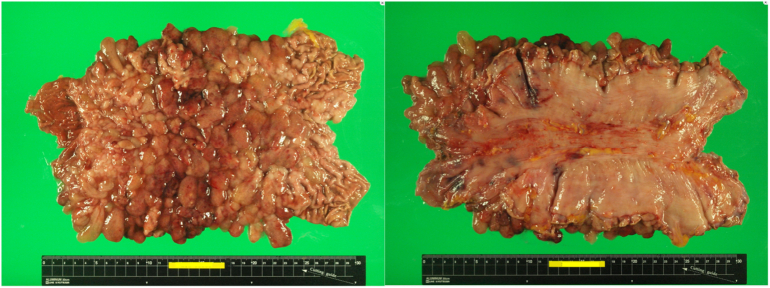
The resected specimen revealed multiple variously sized polyps throughout the stomach except for the lesser curvature and fundus, and the histopathological examination showed that all polyps were hyperplastic with no malignancy. No metastases were observed in any of the 42 dissected lymph nodes.

A 64-year-old woman was admitted to our hospital because she became malnourished due to loss of appetite. She had with no family history of gastrointestinal polyposis and was diagnosed with gastric polyposis and polyp-related anemia eight years previously. She received endoscopic submucosal dissection of early gastric cancer twice in another hospital. (Fig. 1A). Thereafter, the patient received an annual upper gastrointestinal endoscopy and took iron supplements for anemia due to occasional bleeding from polyps. However, the number of polyps increased over time. Laboratory data showed hypoproteinemia, but neither anemia nor elevation of tumor markers (carcinoembryonic antigen, 1.0 ng/ml; carbohydrate antigen 19-9, <2.0 U/ml) was observed. Upper gastrointestinal endoscopy revealed multiple edematous polyps in the entire stomach, and the biopsy sample was histopathologically diagnosed as hyperplastic polyps (Fig. 1B). Enhanced computed tomography showed gastric wall thickening and multiple gastric polyps without lymphadenopathy or distant metastasis (Fig. 2A), and upper gastrointestinal series revealed multiple polypoid lesions widespread throughout the entire stomach except for the lesser curvature and the fundus (Fig. 2B). Colonoscopy showed no specific findings. She had no medical history or physical findings such as skin pigmentation or abnormalities of the hair or nails. She was diagnosed with JPST with a history of gastric cancer resection and underwent laparoscopic total gastrectomy with Roux-en-Y esophagojejunostomy. Although excessive distention and congestion of the stomach were observed intraoperatively, standard laparoscopic surgery could be performed (Fig. 3). The resected specimen revealed multiple variously sized polyps throughout the stomach except for the lesser curvature and fundus, and histopathological examination showed that all polyps were hyperplastic polyps with no malignancy (Fig. 4). The patient was discharged on postoperative Day 10.

## 3 Discussion

We reported a rare case of JPST, for which laparoscopic surgery was performed. JPS is a gastrointestinal polyposis characterized by the development of numerous hamartomatous and nonneoplastic polyps. It was first reported in 1964 as multiple hamartomatous polyps throughout the intestine, with an autosomal dominant inheritance pattern, whereas polyposis restricted to the stomach was first reported in 1975 [5], [6]. JPS has been classified into three types according to the differences in the clinicopathologic characteristics: juvenile polyposis of infancy, juvenile polyposis coli, and generalized juvenile polyposis [7]. The diagnostic criteria of juvenile polyposis include the presence of more than five juvenile polyps in the colorectum, juvenile polyps throughout the gastrointestinal tract, and/or any number of juvenile polyps in a patient with a family history of juvenile polyposis [8]. A fourth type, JPST, characterized by juvenile polyps limited to the stomach at the time of diagnosis, has also been reported. The nomenclature of JPST was first proposed by Watanabe et al. in 1979 [9]. Since then JPST has been reported more frequently in Japan than Western countries. Patients with juvenile polyposis often have autosomal dominant inheritance. In recent studies, in 20–50 % of patients, JPS was caused by germline mutations within the coding sequence of a TGF superfamily gene, namely, SMAD4 on chromosome 18q21.1 or BMPR1A on chromosome 10q22–23 [10]. In the present case, the patient had no family history of juvenile polyposis, and refused a test for germline mutations in the SMAD4 and BMPR1A genes but was diagnosed with JPST based on physical findings and histopathological features.

The association of gastric cancer with JPST is well-known in Japan, and Ishida et al. reported that the lifetime risk is 20 % by the age of 40 years and 61.5 % by 70 years [2]. Moreover, polyposis gives rise to symptoms that are refractory to therapy, such as iron deficiency anemia or hypoalbuminemia. The present strategy for symptomatic or cancer-containing JPST is surgical treatment. In past cases of JPST, recurrence of gastric cancer and polyps in the remnant stomach has been reported [2], [11]. Therefore, total gastrectomy is recommended as standard treatment. It might be worthwhile to perform prophylactic gastrectomy in JPST patients with numerous gastric polyps showing a so-called “coral-like appearance”, since precise endoscopic detection of early gastric cancer may be difficult. Case reports of Japanese JPST patients with gastric cancer searched in PubMed and Igaku Chuo Zasshi (in Japanese) and the present case are presented in Table 1 [3], [12], [13], [14], [15], [16], [17], [18], [19], [20], [21], [22], [23], [24]. There were 17 patients, including 12 females (71 %), with a median age of 44 years (range, 31–65 years). Four patients underwent laparoscopic total gastrectomy, including the patient in the current case. Several reports in Japan have suggested that laparoscopic surgery is a safe and feasible option for patients with juvenile gastric polyposis and is preferable because of better cosmetic effects, especially for young female patients [22], [24]. We demonstrated that standard laparoscopic surgery could be performed nevertheless excessive distention and congestion of the stomach because during manipulation of the stomach, we tried to grasp the stomach in a friendly manner to prevent tissue contusion or damage.

**Table 1:** Case reports of Japanese juvenile polyposis of the stomach with gastric cancer.

Case	Year	Author	Age/sex	Family history of polyposis	Physical findings	Anemia	Hypoproteinemia	Surgical procedure	Laparoscopic surgery
1	1986	Morimoto [11]	36/M	+	+	−	−	DG	−
2	1991	Bizen [12]	65/F	−	−	+	+	DG	−
3	1994	Hamamoto [13]	36/F	−	−	−	−	TG	−
4	1994	Shimono [14]	32/M	−	−	−	−	PG	−
5	1997	Hizawa [10]	45/F	−	Unknown	+	+	PG	−
6	1997	Mitomi [15]	37/F	+	Unknown	+	+	TG	−
7	1997	Mitomi	42/F	+	Unknown	+	Unknown	Polypectomy	−
8	1998	Kitadai [16]	54/F	−	−	+	+	DG	−
9	2008	Tokunaga [17]	31/M	−	Unknown	+	−	DG	−
10	2008	Yamanaka [18]	44/M	−	−	−	−	TG	−
11	2009	Takashima [19]	50/F	+	−	−	+	TG	−
12	2009	Yagi [20]	60/F	−	−	+	+	TG	−
13	2015	Matsuo [21]	37/F	−	−	+	+	TG	+
14	2016	Yasuda [22]	34/F	Unknown	−	+	+	TG	−
15	2018	Yube [23]	48/F	−	−	+	+	TG	+
16	2018	Jogo [3]	63/M	−	−	+	+	TG	+
Our case	2018		64/F	−	−	+	+	TG	+

DG: Distal Gastrectomy, TG: Total Gastrectomy, PG: Partial Gastrectomy.

## 4 Conclusion

We successfully performed laparoscopic surgery to treat a rare case of JPST. JPST has malignant potential of gastric cancer, and JPST patients are needed for careful follow-up.

## Funding

This research did not receive any specific grant from funding agencies in the public, commercial, or not-for-profit sectors.

## Provenance and peer review

Not commissioned, externally peer-reviewed.

## Ethical approval

The study is exempt from ethnical approval in our institution please state this.

## Consent

Written informed consent was obtained from the patient for publication of this case report and accompanying images. A copy of the written consent is available for review by the Editor-in-Chief of this journal on request.

## Author contribution

Misato Ito: performed surgery, conception of report, data collection, data analysis, manuscript writing, revision and submission.

Hisashi Onozawa: performed surgery, data analysis, manuscript writing and manuscript revision.

Masaru Saito: data analysis, manuscript writing and manuscript revision.

Hirofumi Ami: data analysis, manuscript writing and manuscript revision.

Shinji Ohki: performed surgery, data analysis, manuscript writing and manuscript revision.

Yoshihisa Koyama: data analysis, manuscript writing and manuscript revision.

Koji Kono: data analysis, manuscript writing and manuscript revision.

All authors participated in the acquisition, analysis, or interpretation of the data; drafting and revising of the manuscript; and the final approval of the paper. Furthermore, all authors agreed to be accountable for the integrity of the case report and have read and approved the final manuscript.

## Research registration

None.

## Guarantor

Hisashi Onozawa.

## Declaration of competing interest

The authors declare that they have no conflict of interests.
